# Alpha-1 antitrypsin deficiency: an update on clinical aspects of diagnosis and management

**DOI:** 10.12703/b/9-1

**Published:** 2020-10-28

**Authors:** Gabriela Santos, Alice M Turner

**Affiliations:** 1 Pneumology Department, Hospital Garcia de Orta, Almada, Portugal; 2 Institute of Applied Health Research, University of Birmingham, Birmingham, B15 2TT, UK

**Keywords:** alpha-1 antitrypsin deficiency, chronic obstructive pulmonary disease, emphysema, cirrhosis, treatment

## Abstract

Clinical heterogeneity has been demonstrated in alpha-1 antitrypsin deficiency (AATD), such that clinical suspicion plays an important role in its diagnosis. The PiZZ genotype is the most common severe deficiency genotype and so tends to result in the worst clinical presentation, hence it has been the major focus of research. However, milder genotypes, especially PiSZ and PiMZ, are also linked to the development of lung and liver disease, mainly when unhealthy behaviors are present, such as smoking and alcohol use. Monitoring and managing AATD patients remains an area of active research. Lung function tests or computed tomography (CT) densitometry may allow physicians to identify progressive disease during follow up of patients, with a view to decision making about AATD-specific therapy, like augmentation therapy, or eventually surgical procedures such as lung volume reduction or transplant. Different types of biological markers have been suggested for disease monitoring and therapy selection, although most need further investigation. Intravenous augmentation therapy reduces the progression of emphysema in PiZZ patients and is available in many European countries, but its effect in milder deficiency is less certain. AATD has also been suggested to represent a risk factor and trigger for pulmonary infections, like those induced by mycobacteria. We summarize the last 5–10 years’ key findings in AATD diagnosis, assessment, and management, with a focus on milder deficiency variants.

## Introduction

Alpha-1 antitrypsin deficiency (AATD) is an autosomal co-dominant disease, usually underdiagnosed owing to its variable penetrance and clinical heterogeneity. The alpha-1 antitrypsin (AAT) protein is encoded by the *SERPINA1* gene on chromosome 14, and its main function is to inactivate neutrophil elastase (NE) upon insult to the lungs, such as smoking. In its absence, there is an imbalance of proteinases and anti-proteinases, which leads to the progression of emphysema and deterioration of lung function, resulting in chronic obstructive pulmonary disease (COPD). In some mutations, polymerization of AAT in alveolar macrophages and the presence of pro-inflammatory AAT polymers, previously reported to be obtained in bronchoalveolar lavage in PiZZ patients, contribute to the pathogenesis in AATD lungs^1^. This mini-review summarizes key findings in this disease’s diagnosis, assessment, and management from the last 5–10 years.

## Which patients develop clinically relevant disease?

A number of genetic mutations cause AATD. It has long been accepted that the Z allele, and in particular the PiZZ genotype, is linked to emphysema and early onset COPD^2^. There is also limited evidence that patients with null mutations have worse prognosis^3^.

In recent years, there has been growing interest in the relative risk conferred by genotypes causing milder deficiency, such as the S allele. The S protein forms fewer polymers than does the Z protein; therefore, it is retained less within hepatocytes and leads to less endoplasmic reticulum protein overload. Consequently, the S allele is only a minor risk factor or co-factor for cirrhosis in specific subpopulations such as chronic alcohol abusers. On the other hand, alcohol stimulates AAT production in hepatocytes, which may aggravate liver function in carriers of a single abnormal allele, in particular in carriers of the more pathogenic Z allele^4^. Circulating AAT is inversely proportional to the amount of liver polymerization/retention of each type of AAT; Table 1 shows some of the milder deficiency genotypes, levels, and risks of disease.

**Table 1.** Milder deficiency genotypes, alpha-1 antitrypsin (AAT) levels, and risk of disease.

Genotype	*Average AAT level^13^		Risk of disease	References
SZ	9–15 µM	45–80 mg/dL	COPD (related to smoking or occupational exposure; 3x > PiMM) Lung function decline (DLCO > FEV1) Apical emphysema dominance, with less severe disease than PiZZ Risk factor for chronic liver disease	5,9–12
MZ	13–23 µM	66–120 mg/dL	Higher risk of emphysema compared to PiMM Increased risk of COPD in smokers/ex-smokers Lung function decline (FEV1 > DLCO) Higher transaminase levels Modifier of chronic liver disease (alcoholic cirrhosis, non-alcoholic liver disease, or cirrhosis)	4,9,14–17
SS	14–20 µM	70–105 mg/dL	Obstructive lung disease (COPD; asthma) Minor risk liver cirrhosis in alcohol abusers	4,18
MS	19–35 µM	100–180 mg/dL	Without lung or liver risk disease	13

AAT, alpha-1 antitrypsin; COPD, chronic obstructive pulmonary disease; DLCO, diffusing capacity of lung for carbon monoxide; FEV1, forced expiratory volume in 1 second *Serum levels given are measured using commercial standard (mg/dL) and the purified standard (µM)

Whilst their milder genetic profile when compared with PiZZ makes PiSZ, SS, and MZ patients less likely to develop adverse effects linked to AATD, such genotypes are much more prevalent than ZZ in the world^5–7^, and in the presence of unhealthy behaviors they become big risk groups for the development of lung disease. This enhances public health need to increase diagnosis and implement preventive measures in these patients^7,8^.

### SZ genotype

More than 700,000 PiSZ patients have been reported in Europe^7^. The major clinical risk in PiSZ is the development of COPD, which is three times higher compared with PiMM^9^, less so in never-smoking patients^10^. When PiSZ patients develop emphysema, usually it has an apical dominance^5^; physicians’ cognitive bias to screen for AATD mainly in basal emphysema may exclude them from testing and follow-up, thus leading to a greater proportion of undiagnosed patients relative to PiZZ. Reversibility has also been observed in a large number of patients, which is frequently associated with more severe airflow obstruction^10^. Abnormalities in forced expiratory volume in 1 second (FEV1) are associated with basal-predominant emphysema, usually present in PiZZ, while abnormality in diffusing capacity of lung for carbon monoxide (DLCO) is associated with upper-zone emphysema^11,12^, which is often seen in PiSZ patients. Since these types of emphysema may be driven by different mechanisms^2^, we can speculate that the pathophysiology of emphysema differs between PiSZ and PiZZ genotypes such that therapy applicable to PiZZ cannot be assumed to be effective in PiSZ. Although disease progression in PiSZ patients has been reported to be similar to that in PiZZ patients, the evidence for this is inconsistent^10^. Furthermore, the survival rate seems to be better in PiSZ; the decline in FEV1 can be up to 169% faster in PiSZ when compared with PiMM but may not be a good predictor of survival^19^. It is possible that computed tomography (CT) densitometry or DLCO would be more informative regarding survival given that upper zone density decline is relevant to mortality^11^ and is common in PiSZ patients.

Just like in lung disease, PiSZ patients express a milder form of liver disease than PiZZ patients, since liver toxicity is proportional to the amount of retained protein (PiZZ > PiSZ). The Z allele in PiSZ genotype confers an increased risk for cirrhosis in chronic metabolic injury (six times higher), such as in non-alcoholic fatty liver disease (NAFLD) and chronic alcohol abuse^4^. The association between PiSZ heterozygosity and risk of developing other complications of AATD such as panniculitis and granulomatosis with polyangiitis is controversial but smaller than PiZZ homozygosity^7^.

### SS genotype

PiSS genotypes are rarely diagnosed in clinical practice. Although the S allele is more common than the Z allele, interestingly, PiSS is not as commonly found as other genotypes^6,20,21^. For that reason, it is difficult to get accurate results regarding clinical phenotype. However, it has been noticed in a small cohort that COPD and asthma had a higher prevalence than expected^18^. As for liver disease, it remains undetermined if there is any clinical association, although the incidence was higher than predicted in one cohort study^18^.

### MZ and MS genotypes

PiMZ and PiMS are the most frequent AATD genotypes^6,20,21^. PiMS is the least studied group, since many assume that it has no clinical relevance, given that AAT levels are close to normal. Limited evidence suggests that when smoking history is controlled, this group is not at risk for COPD when compared with the general population^9^. The PiMZ genotype is especially important when it comes to current or ex-smokers, as their risk for COPD becomes similar to that of PiSZ^14^. Furthermore, decline of lung function and an increased risk for emphysema development have been shown^15^.

Whether or not PiMZ individuals are at risk of developing liver disease is controversial. The presence of the Z allele was associated with higher transaminase levels, increased risk of progression of alcoholic cirrhosis and non-alcoholic liver disease, higher rates of decompensation of cirrhosis, and increased risk of liver transplantation^16^. As for the risk of liver cancer in PiMZ individuals, this is even more controversial, with some studies suggesting a risk for cholangiocarcinoma^22^ and others reporting no association at all^23^. The presence of the Z allele might enhance susceptibility for carcinogenesis, as pre-neoplastic and neoplastic lesions were largely found to arise from PAS-D-devoid areas in PiZ mice^24^, similar to lesions found in AATD patients with hepatocellular carcinoma^23^. Further studies are still needed to confirm these assumptions.

### Health behaviors

Health behaviors also play an important part in the presentation and management of patients with AATD. In order to present clinically with significant disease, milder deficiency genotypes require more intense environmental exposures to manifest. A summary of these health behavior differences is presented in Table 2.

**Table 2.** Health behaviors in individuals with milder genotypes of alpha-1 antitrypsin deficiency.

Genotype	Health behaviors	References
SZ	More likely than PiZZ to exhibit unhealthy behaviors (sedentary lifestyle, overweight, active smokers) Longer periods of smoking and higher number of packs smoked per day than PiZZ More frequent exacerbations and hospitalizations than PiZZ More visits to primary and lung physicians than PiZZ A lesser proportion of PiSZ reported to consume alcohol compared to PiZZ	5,8
MZ	Worse health behavior than PiZZ in general Worse health behavior prior to developing lung disease Broader pattern of unhealthy behavior prior to the development of lung disease (poor exercise habits, active smoking); may persist after the diagnosis Less engaged to proceed with smoking cessation-related behaviors than PiSZ or PiZZ	8
SS	Unknown relationship with lung disease Minor risk factor for developing cirrhosis only in alcohol abusers	4

Smoking cessation is the most important protective measure in AATD, even though there are studies reporting a minor effect when comparing PiSZ with PiZZ. Nevertheless, a faster rate of decline in lung function has been observed in both genotypes, which indicates that tobacco cessation must be a priority^25,26^. PiSZ patients exhibit a lower risk of lung disease and are less susceptible to smoking effects when compared with PiZZ patients^10^; however, because of their higher AAT levels, they may have less concern that their genotype presents a risk of disease, prompting them to unhealthy behaviors^5,8^. Emphasizing smoking cessation and behavioral interventions among PiSZ is likely to be highly beneficial, as they have an increased risk of developing COPD when compared to PiMM smokers^14^. Regardless of genotype, additional education about moderation of alcohol consumption should be considered because of the increased risk of liver disease among individuals with AATD. Reduction of harmful inhaled substances from occupational exposure should also be advised.

## Recommendations for AATD diagnosis

AATD testing is recommended for all adults with emphysema, COPD, or asthma, whenever airflow obstruction is present or incompletely reversible, after optimized treatment with bronchodilators^14,25,27,28^. Other rarer forms of AATD might be present, so unexplained bronchiectasis, granulomatosis with polyangiitis, necrotizing panniculitis, and liver disease of unknown etiology should also prompt further AATD testing^14,25,27,28^. Once the diagnosis is made, familial testing is advocated, since AATD is a heritable disease.

**Figure 1.**
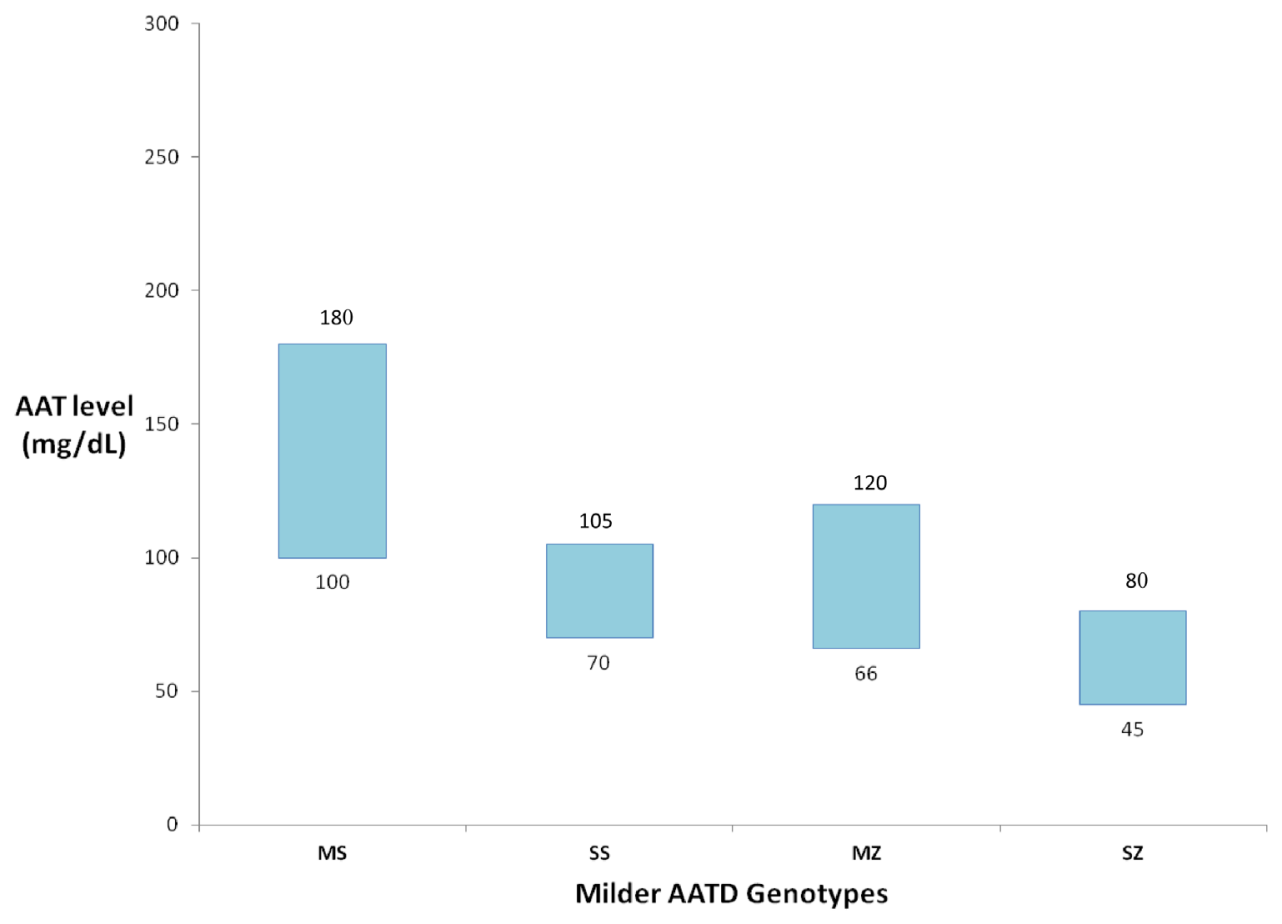
Serum alpha-1 antitrypsin (AAT) levels associated with milder AAT deficiency (AATD) genotypes (MS, SS, MZ, and SZ)

AAT levels alone are inaccurate for identifying these patients since equivalent AAT levels could represent different milder AATD genotypes^13^, as demonstrated in Figure 1. Confirmatory testing, through phenotyping and genotyping, are strongly recommended to identify normal, deficient, or non-functioning alleles, or even rarer AAT alleles, which otherwise would go unrecognized^14,27,28^.

## New diagnostic modalities

A delay in diagnosis has been associated with worsened clinical status^29,30^, so there has been a focus on ways to make diagnostic testing easier and more efficient. AATD screening usually starts by measurement of the level of AAT in the blood and, if it is low, followed by phenotype or genotype for definitive confirmation. Phenotyping refers to testing the speed of protein migration by isoelectric focusing, whilst genotyping is usually done for specific mutations (usually for the S and Z mutations). Newer approaches which allow home testing or testing in primary care are desirable and include the Alphakit® Quickscreen (Diagnostic Grifols, Barcelona, Spain) for the identification of the Z protein using lateral-flow paper-based technologies^31^. A positive result should prompt further investigation. A limitation of this approach is that a negative result (absence of Z protein in blood) may lead to underdiagnosis of non-Z AATD genotypes. A newer Luminex-based algorithm capable of detecting 14 different AATD mutations simultaneously, compared to the two traditional mutations (S and Z), in a shorter time has also been developed^32^. This can be performed from drops of blood from a fingerstick or a buccal swab and covers >98% of mutation combinations known to cause AATD. Table 3 shows the methods of diagnosis reported to date.

**Table 3.** Methods of diagnosis for alpha-1 antitrypsin deficiency (AATD).

Test	Advantages	Disadvantages	References
*Serum AAT tests*			
Nephelometry	Good reliability Inexpensive Standard method	Does not reliably detect heterozygotes	28,33
Radial immunodiffusion	Inexpensive None above nephelometry	Overestimates the concentration of AAT Inaccurate; not in use	28,33
Rocket electrophoresis	Inexpensive None above nephelometry	Inaccuracy and low sensitivity; not in use	28,33
*Phenotyping and genotyping*			
Point of care detection of serum Z protein (Alphakit Quickscreen)	Detects Z allele homozygotes or heterozygotes Exclusion of non-Z AATD in primary care and in the overall chronic obstructive pulmonary disease population, with low pre-test probability Widely available and easy to interpret Small samples needed Cost-effective	Low negative predictive values in a population with a very high pre-test probability False negatives in PiMZ samples	31,33
Isoelectric focusing (IEF) method	Detects S and Z alleles and rare variants (F, I, and P) Identifies heterozygotes Highly specific and rapid Simple to perform Useful in screening programs	Null (Q0) mutations or M-like alleles are not detectable Interpretation of rare alleles can be difficult No longer regarded as standard for phenotyping	33
PCR-based tests	Detects the Mmalton allele Molecular diagnosis of S and Z allele	Null (or Q0) mutations are not detectable Requires specific primers for each allele	33
Luminex technology	Detects 14 AATD mutations simultaneously Short time to conduct testing Cost-effective Detects abnormalities across the entire genome using less DNA	Requires sophisticated bioinformatics systems to analyze and clinically interpret the data	32,33
*Gene sequencing*			
Sanger method	Detects mutations caused by a variety of different mechanisms, including deletions, insertions, point mutations (silent, nonsense, and missense), and frameshift mutations Permits sequencing of introns	Can be expensive Not available in every hospital Requires sophisticated bioinformatics systems to analyze and clinically interpret the data	34

## Clinical features of AATD

### Pulmonary involvement

**Figure 2.**
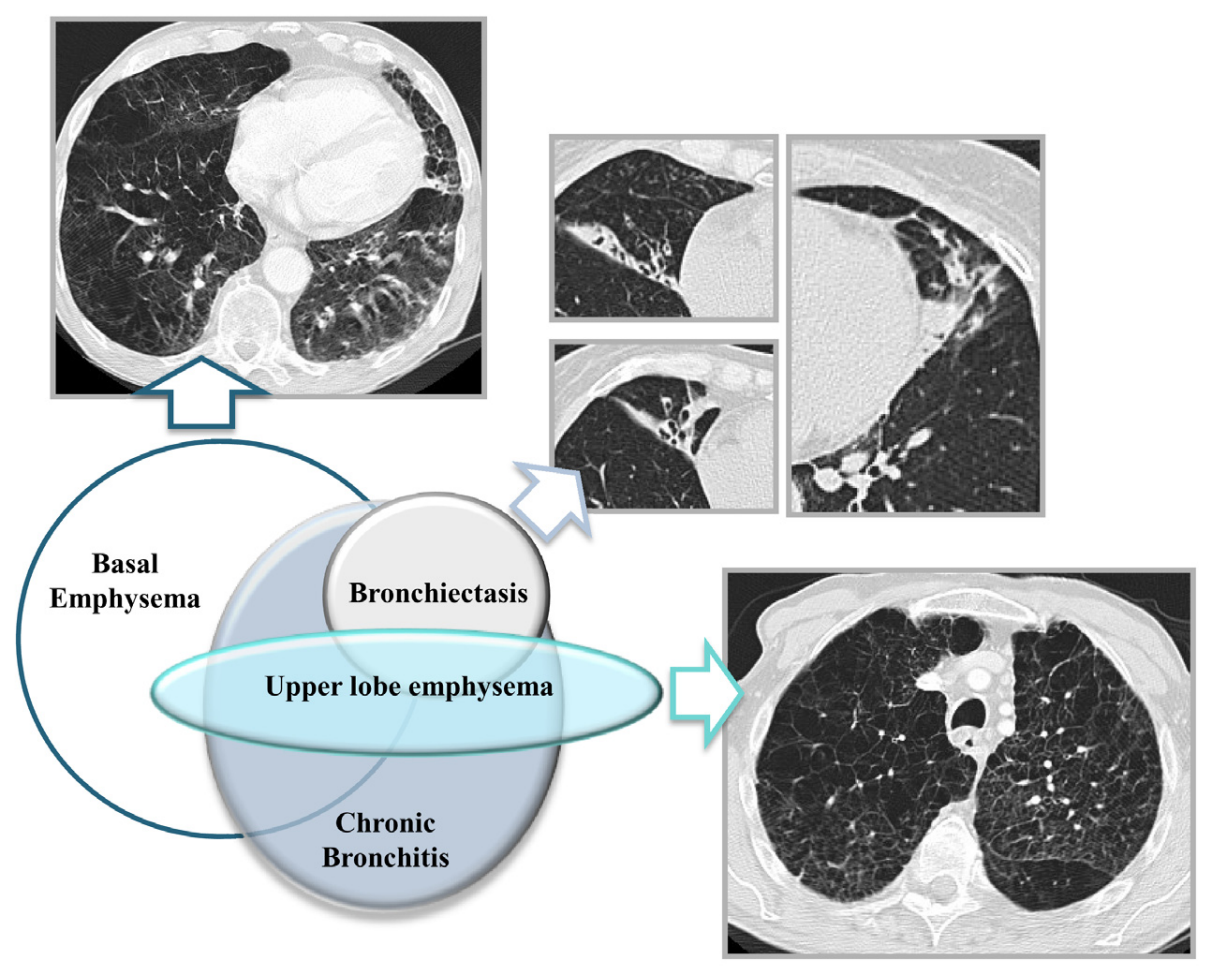
Pulmonary features from alpha-1 antitrypsin deficiency (AATD) are shown in the Venn diagram, representing the relationships between them. Most patients will have predominant basal emphysema, and a small proportion, simultaneously or not, has upper zone emphysema. Bronchiectasis is less common in AATD and often associated with emphysema. Chronic bronchitis features might be present in AATD patients even before major structural changes are observed. Written informed consent was obtained from the patient/patient’s family for the use and publication of these clinical images.

Emphysema and COPD are the main clinical features of AATD; severity depends on genotypes and health behaviors (discussed above). AATD lung disease is characterized by basal pan-lobular emphysema at an early age, though a range of other phenotypes have been recognized (Figure 2). Reversibility of airflow obstruction is observed in up to 80% of AATD patients^2,35^. This has prognostic impact, since the degree of reversibility associates with rapid decline of lung function^36^. Chronic bronchitis (CB) affects approximately 40% of patients with AATD^37^. CB, as part of the spectrum of neutrophilic inflammation in the lungs, might be one of the clinical features that should draw attention to AATD diagnosis^2^. Nevertheless, clinical heterogeneity makes AATD a challenging diagnosis.

The relationship between asthma and AATD is unclear, although it has been proposed^38^ that patients tested and diagnosed with AATD at an early age are more likely to be labeled as asthmatic^28^. This uncertainty, and the presence of asthma symptoms, with fixed or reversible obstruction in lung function in significant numbers of AATD patients, is a factor behind the recommendation to test for AATD in a wide range of respiratory patients^39^. Allergic asthma is usually more common in younger AATD patients, and AAT serum levels have been shown to be lower in asthmatic carriers of a Z allele^40^. However, no significant association was observed between common *SERPINA1* SNPs and the risk of developing school‐age asthma, the presence of a deficient allele (S or Z) did not affect the risk of wheezing in childhood and further development of asthma in adolescence^41^, and no association was made between AATD genotypes or lung function severity with allergic asthma severity^40^. Future research is needed, as there are inconsistent data regarding an association between AATD and asthma.

Bronchiectasis is found in many AATD patients, although it is usually encountered in patients who already have emphysema, suggesting that there is a shared pathophysiological process underway^2^. Bronchiectasis may also present as part of pulmonary Langerhans cell histiocytosis (PLCH). PLCH is strongly linked with cigarette smoking, manifests in young adults, and is characterized by the presence of polycystic lung lesions. It has been speculated that AATD patients might be at a greater risk for developing PLCH, as cystic pulmonary lesions have been observed^42,43^.

The pulmonary microbiota in AATD patients differs from that of usual COPD smoking patients. AATD patients on augmentation therapy (AT) have lower sputum neutrophils and a lower specific bacterial load (*Moraxella catarrhalis* and *Streptococcus pneumonia)*^44^. Among bronchiectasis patients, the risk of non-tuberculous mycobacteria (NTM) infection seems to be higher in AATD patients when compared to primary ciliary dyskinesia and common variable immunodeficiency^45^, perhaps because AAT inhibits rapid growth of mycobacterial infection in macrophages, thus enhancing macrophage immunity against NTM^46,47^. A potential link between AATD and invasive infections, like invasive pulmonary aspergillosis, has also been postulated^48^.

### Extrapulmonary involvement

**Figure 3.**
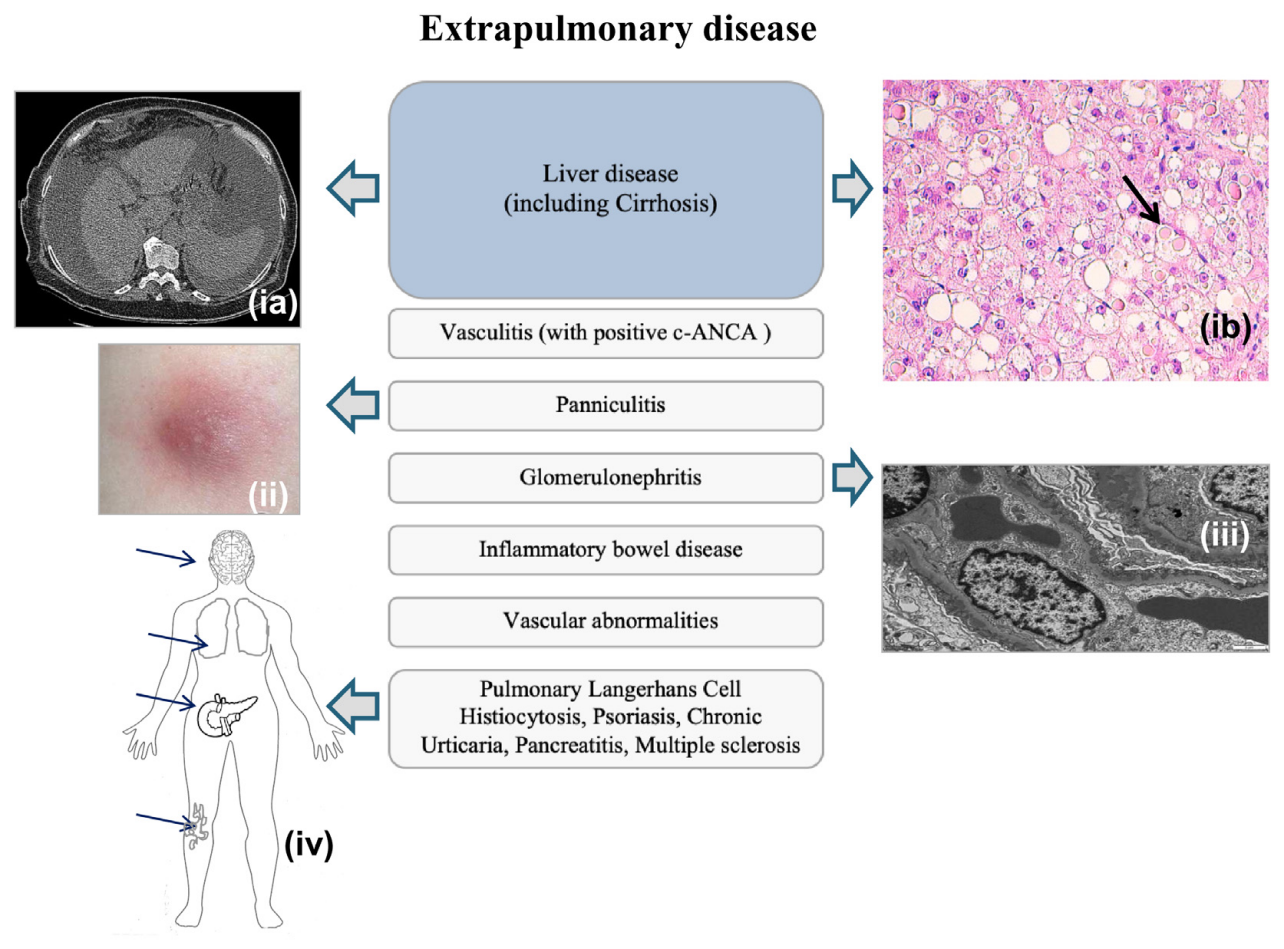
Alpha-1 antitrypsin deficiency (AATD) extrapulmonary manifestations. AATD extrapulmonary manifestations consist mainly of liver disease (including cirrhosis [image ia: computed tomography {CT} scan; ib: periportal hepatocytes with numerous eosinophilic globular inclusions which were Periodic Acid-Schiff stain {PAS} and AAT positive]). In a smaller proportion, there may be vasculitis, panniculitis (ii), and glomerulonephritis (iii). Very rarely, pulmonary Langerhans cell histiocytosis, psoriasis, chronic urticaria, pancreatitis, and multiple sclerosis have been reported to be associated with AATD (iv). Written informed consent was obtained from the patient/patient’s family for the use and publication of the clinical image (ia). Image (ii) was adapted from Robert A. Stockley and Alice M. Turner^2^. Image (ib) and (iii) were taken from the laboratory at Hospital Garcia de Orta with permission from Dr. Maria Brito from the Pathology Department. Image (iv) is an original image produced by the authors for this review article.

Diseases such as panniculitis and vasculitis are observed, albeit rarely. Necrotizing panniculitis and systemic vasculitis with positive c-ANCA should prompt testing for AATD, since an association between them has been established^28^. Other reported associations of AATD from cases and small cohort studies include inflammatory bowel disease, glomerulonephritis, rheumatoid arthritis, fibromyalgia, vascular abnormalities (fibromuscular dysplasia of the arteries, abdominal and brain aneurysms, and arterial dissection), psoriasis, chronic urticaria, pancreatitis, and multiple sclerosis (Figure 3). Although these are rare associations, they are plausible, since AAT is anti-inflammatory and immunomodulatory^47,49^; thus, in AATD, enhanced risk of inflammatory and autoimmune diseases could occur. It has even been proposed that AT could help to prevent these issues, though it is controversial^50^.

## Monitoring patients with AATD

### Imaging markers

Usually lung function is used to evaluate the progression and deterioration of AATD^14^. The measurement of pulmonary emphysema through CT densitometry has become more common in recent research. CT density has been associated with clinically relevant parameters, such as FEV1 and quality of life (Saint George’s Respiratory Questionnaire [SGRQ]), and has a clear and consistent relationship with mortality^51^ in COPD, which showed that density could be a valid surrogate outcome for disease severity. Use of CT densitometry in disease monitoring has been vital in proving an effect of AT in emphysema^10^, and lower CT density has also been related to mortality in AATD patients with basal emphysema, while FEV1 and DLCO alone have a weaker relationship^11^. This suggests that densitometry may be a useful clinical tool in AATD; however, clinical heterogeneity, lack of longitudinal data, and inter-individual lung volume variation are some of the limitations in the wide adoption of this technology.

### Biological markers

Desmosine and isodesmosine (lung elastin degradation products usually elevated in COPD patients but also in AATD patients) were reduced after long-term intravenous AT and possibly with nebulized therapy^52^. The plasma degradation product of fibrinogen (Aα-Val360) was a disappointing marker, lacking a linear progression with time when considering its relationship between disease activity and severity, although it does reduce with augmentation^53^. The presence of elevated free light chains could also play a role in risk stratification in AATD patients, since they independently predict mortality in patients with severe AATD and usual COPD. At present, they are a more important pathogenic theme in usual COPD, but contribution to immune activation within the disease process in AATD is not excluded^54^. More recently, complement component C3d was proposed, since it correlates with both radiographic emphysema and severity of the emphysema in AATD, but not in usual COPD; also, in PiZZ AATD after intravenous AT, AAT disrupts C3 activation, thereby decreasing C3d plasma levels. The role of C3d in AATD is still unknown; however, a potential role for the complement system is emerging in the pathogenesis of emphysema^55^. Finally, interleukin (IL)-27, a cytokine released by macrophages and neutrophils, has been proposed, as its levels appear to reflect sputum neutrophilia and bacterial load, postulating a relationship between IL-27 and bacterial survival, and correlate with FEV1. Further investigations are needed to establish the relationship among neutrophil recruitment, IL-27 production, and bacterial load in AATD^44^.

## Treatment and management of patients with AATD

### General COPD treatment

Most AATD patients’ management is based on COPD prevention and maintenance therapy. It is important to initiate and maintain bronchodilator therapy, with a good inhaler technique, such as long-acting β-adrenergic receptor agonists (LABA) and long-acting muscarinic receptor agonists (LAMA)^25^. It is conceivable that targeting pro-inflammatory pathways with inhaled corticosteroids (ICS) would be more beneficial in AATD patients, since exacerbation rates are higher and longer than in usual COPD^56^, but this remains unproven. Evidence is present that the response to ICS in AATD is associated with blood eosinophil count^57^, as in usual COPD, implying that a blanket approach would be inappropriate. Macrolides reduce the risk of exacerbations in usual COPD^58^. We might speculate that there would be the same effect on AATD patients with COPD, although data are lacking in this area. In severe AATD patients with established emphysema, AT should also be offered, according to guidelines^14,28^.

Influenza and pneumococcal vaccination should occur, as AATD patients have a high susceptibility for lower respiratory tract infections^44,56,59^. Clinical benefits of pulmonary rehabilitation (PR) have been questioned in AATD patients, as unfavorable muscle response to exercise has been proposed^60^. Nevertheless, PR has improved health status, exercise tolerance, and quality of life, all problems that AATD patients experience, thus is reasonable to recommend. In cases of severe chronic hypoxemia at rest, long-term oxygen therapy improves survival, and if chronic hypercapnia is also present, long-term non-invasive ventilation might decrease hospitalizations and mortality, as in usual COPD^58^. Palliative approaches should always be initiated in cases of refractory symptoms.

Although recommendations for general treatment in AATD are based on usual COPD management, the majority of COPD pharmacotherapy clinical trials exclude these patients^58,61–65^.

### Augmentation therapy

The use of AAT-AT is highly variable throughout Europe owing to variable health policies, product registration, and reimbursement issues. France and Germany have the most patients receiving AAT-AT (around 60%), whereas in Spain only approximately 20% of patients are receiving treatment^25^. Several countries such as the UK do not cover AAT-AT. AAT-AT has produced beneficial consequences, like ameliorating lung function decline and emphysema progression, prolonging survival, and delaying the decline in quality of life, especially in severe AATD, i.e. in ZZ or Z null patients^14,56,66–68^. Controversy remains over the effect on exacerbations, since a meta-analysis of randomized controlled trials (RCTs) revealed a small statistically significant increase in annual exacerbations (0.29/year) on AAT-AT^56^, shown in Table 3. However, evidence of this is inconsistent. AAT-AT was related to a significant reduction in exacerbation rate^69^ and a reduction in exacerbation severity^70^ in cohort studies. The potential benefits of AAT-AT in PiZZ patients are summarized in Table 4.

**Table 4.** Potential benefits of alpha-1 antitrypsin augmentation therapy (AAT-AT).

Clinical feature	Effect of AAT-AT versus non-treated patients	Evidence type/average follow-up
CT density	Slower rate of emphysema progression (0.79 g/L/year [95% CI 0.29–1.29; *P* = 0.002])	Meta-analysis^56^ (until 2017)
	Decreased rate in emphysema progression (0.74 g/L/year [95% CI 0.06–1.42; *P* = 0.03])	RCT^74^ (4.6-year approximately)
	Reduction in decline rate of emphysema (–1.26 g/L/year [standard error 0.29; *P* = 0.001])	Open label extension^75^ (4.6-year approximately)
	Smaller change in lung density in treated group (–4.08 g/L treated versus –6.38 non-treated) Reduction in lung density (2.30 [95% CI 0.67–3.93; *P* = 0.006]) in 2.5 years	Combined studies^70^ (2.5-year)
Lung function	FEV1% predicted: 0.56% predicted/year (95% CI 1.14–0.29; *P* = 0.20)	Meta-analysis^56^ (until 2017)
	FEV1% predicted: 47.4 ± 12.1% treated versus 47.2 ± 11.1% non-treated	RCT^74^ (4.6-year)
	FEV1: 1.25 L treated versus 1.19 L non-treated (*P* <0.05)	Observational, retrospective^69^ (3-year)
	FEV1% predicted: 37 ± 18% treated versus 74 ± 35% non-treated	Re-analysis AATD registry group data^76^ (8-year)
	FEV1% predicted: 48 ± 16.4% treated versus 47.9 ± 18.6% non-treated	Combined studies^70^ (2.5-year)
	Improvement in DLCO (0.11 [–0.33–0.11; *P* = 0.34])	Meta-analysis^56^ (until 2017)
	Improvement in DLCO (58.9 ± 26.3 treated and 69.1 ± 69.2 non-treated)	Observational, retrospective^69^ (3-year)
Exacerbations	0.29/year (0.02–0.54; *P* = 0.02) exacerbations; small but significant increase in annual exacerbation rate on treatment group	Meta-analysis^56^ (until 2017)
	Increased risk of exacerbation in non-treated patients (1.4- to 4.2-fold; *P* <0.05)	Observational, retrospective^69^ (3-year)
Health status	Increased deterioration in SGRQ on placebo (0.83 [–3.55–1.89; *P* = 0.55])	Meta-analysis^56^ (until 2017)
Mortality	Improved survival on treatment group	Re-analysis AATD registry group data^76^ (8-year)

alpha-1 antitrypsin deficiency, AATD; CI, confidence interval; computed tomography, CT; DLCO, diffusing capacity of lung for carbon monoxide; FEV1, forced expiratory volume in 1 second; RCT, randomized controlled trial; SGRQ, Saint George’s Respiratory Questionnaire.

It should be noted that most of the evidence relates to PiZZ patients. In addition, most guidelines recommend the presence of emphysema, a specified level of FEV1, and a specific level of AAT, which excludes almost 90% of PiSZ patients^10,28,71^. While there may be a small proportion of PiSZ patients who might benefit from AAT-AT, such as those rapidly declining with AAT levels below threshold limit (11 µM), scientific evidence supporting clinical efficacy continues to be vague. In several European countries, health authorities have funded AT despite a lack of evidence of benefit in PiSZ patients. Close follow-up in rapid decliners and a wait-and-see approach should be maintained, restricting therapy to those most at risk and aiming for a better quality of life for the patient.

Although dosage has been established at 60 mg/kg/week, it has been proposed that doubling the dosage (120 mg/kg/week) could be even more beneficial because it leads to serum trough AAT levels at physiologic values. A more pronounced impact on slowing disease progression, an overall reduction of anti-proteolytic effect, with significant reductions of collagenase (matrix metalloproteinase-1 [MMP1]) and gelatinase (MMP9), and a reduction in inflammatory effects, namely a significant decrease in IL-10, an anti-inflammatory cytokine important in limiting local host immune responses, have been reported^72^. Further studies are still required.

### Lung volume reduction and transplantation

More invasive approaches like lung volume reduction surgery (LVRS) can be offered; LVRS has demonstrated benefits in AATD, but it seems to be inferior when compared with usual COPD, since it has a higher short-term mortality^56^. Bronchoscopic interventions, like endobronchial valves and lung coils, can improve health status and lung function at least for 6–12 months following treatment, although a small study has reported a 2-year beneficial period^56^. Although these approaches are possible in selected patients, their long-term benefits remain to be elucidated. In addition, the usual approach targeting apical disease is not very useful for patients with AATD; perhaps newer coil procedures may be more useful, though data are lacking to prove this at present.

AATD patients represent 5% of lung transplants performed worldwide, but outcomes and survival rates in a post-transplant phase are still unknown. A recent retrospective study evaluated the incidence of complications and survival of AATD recipients with a control group of COPD recipients^73^. They observed (i) early bronchial anastomotic complications and (ii) late bowel complications. Anastomotic complications with dehiscence were seen only in AATD patients who were under AT and discontinued it before the transplant. This was associated with a probable rebound phenomenon characterized by increased neutrophil activity on bronchoalveolar lavage. Conversely, AATD patients who did not receive AT had better lung outcomes and greater survival rate. Bowel inflammation associated with ischemia was observed too but only in AATD recipients, not in COPD recipients^73^. Since a probable link between timing of withdrawal of replacement therapy in AATD patients and anastomotic complications might be present, new strategies should be considered when referring these patients for lung transplant. Nevertheless, significant health status benefits have been generally observed after transplant, indicating that it is appropriate when quality of life is poor^56^.

When comparing survival rates after lung transplantation, between AATD recipients and usual COPD, no difference in long-term survival was observed in the majority of the studies, albeit AATD patients are usually younger and have fewer comorbilities^56^. Only two studies have reported otherwise, with a 10-year survival superior in COPD patients then in AATD patients^77,78^.

### Products in development

A recent review has examined the different experimental approaches being pursued in trials in AATD^79^, and covering them in detail is beyond the scope of this review. These approaches are summarized in Table 5.

**Table 5.** Active and unpublished clinical trials in alpha-1 antitrypsin deficiency (AATD).

Treatment approach	Phase/trial identifier	Results to date/primary outcome	References
Small molecules	Phase II NCT04167345	Recruiting Primary outcome: evaluate the efficacy, safety, and pharmacokinetics of VX- 814 in PiZZ subjects	80
*AAT-AT*			
AAT-AT (i.v.) (60 versus 120 mg/kg)	Phase III NCT01983241	Recruiting Primary outcome: change from baseline in whole lung PD15 (15th percentile point) determined by CT lung densitometry	81
AAT-AT (i.v.)	Phase III NCT02525861	Active, not recruiting Primary outcome: evaluate the safety and potential immunogenicity and assess the effects of alpha-1 proteinase inhibitor therapy on the levels of AAT and various biomarkers in the epithelial lining fluid	82
AAT-AT (i.v.)	Phase III NCT02722304	Terminated early owing to low/slow enrollment Primary outcome: rate of change in lung density based on group 1 (ARALAST NP) versus placebo and all alpha-1 proteinase inhibitor recipients versus placebo	83
AAT-AT (i.v.)	Phase I–II NCT02870309	Completed Primary outcome: safety of 60 mg/kg alpha-1 MP assessed by AEs, SAEs, discontinuations due to AEs or SAEs, and COPD exacerbations Results: the pharmacokinetics and safety of alpha-1 MP in Japanese subjects with AATD were consistent with the alpha-1 MP profile in non-Japanese subjects	84
AAT-AT (i.v.)	Phase I–II NCT02870348	Active, not recruiting Primary outcome: safety of 60 mg/kg alpha-1 MP as assessed by AEs and SAEs, discontinuations due to AEs or SAEs, and COPD exacerbations	85
AAT-AT (i.v.)	Phase II NCT03385395	Withdrawn Non-inferiority of OctaAlpha1 compared to alpha-1 proteinase inhibitor in terms of the serum trough levels at steady state	86
AAT-AT s.c.	Phase I NCT03362242	Active, not recruiting Primary outcome: number of participants with AE possibly or probably related to treatment	87
Inhaled AAT-AT	Phase III NCT04204252	Recruiting Primary outcome: FEV1 post bronchodilator	88
*NE inhibitors*			
Oral NE	Phase II NCT03636347	Recruiting Primary outcome: change from baseline on blood biomarkers of neutrophil elastase activity (plasma desmosine/isodesmosine)	89
Oral NE	Phase II NCT03679598	Recruiting Primary outcome: evaluate change in plasma desmosine/isodesmosine and emergent adverse events	90
Nebulized hyaluronan	Phase II NCT03114020	Terminated (enrollment stopped 18 November 2019 because of slow enrollment) Primary outcome: measurement of sputum, plasma, and urine concentrations of desmosine and isodesmosine using hyaluronic acid inhalation versus placebo	91
*Gene therapy*			
AAVrh.10 vector-AAT (i.v.)	Phase I–II NCT02168686	Completed Primary outcome: number and proportion of subjects experiencing adverse effects using i.v. AAV gene transfer vectors expressing human AAT	92
rAAV2-CB-hAAT vector (i.v.)	Phase I NCT00377416	Active, not recruiting Primary outcome: presence of rAAV2-CB-hAAT vector in blood and semen using recombinant AAV vectors	93
rhAAT-Fc-AAT (i.v.)	Phase I NCT03815396	Active, not recruiting Primary outcome: frequency and severity of AEs using open-label single and dose-escalation administrations of Fc fusion protein (rhAAT-fc)	94
rAAV2-AAT (intramuscular)	Phase I	Terminated (rise in anti-AAV titers and insufficient AAT levels)	95
rAAV1- AAT (intramuscular)	Phase I	Terminated (subtherapeutic but sustained AAT response, undesirable immune reaction)	96
Other: oral	Phase II NCT03008915	Active, not recruiting Primary outcome: pulmonary microvascular blood flow using aspirin versus placebo in AATD patients	97
*AATD liver trials*			
RNAi (s.c.)	Phase I NCT02503683	Terminated (observation of low incidence of asymptomatic, transiently elevated liver enzymes in a subset of study subjects) Primary outcome: the safety of alpha-1 proteinase inhibitor evaluated by the proportion of subjects experiencing AEs, SAEs, and AEs leading to study drug discontinuation	98
siRNA (s.c.)	Phase II–III NCT03945292	Recruiting Primary outcome: evaluate the safety, tolerability, and effect on liver histology parameters with administration of the investigational product	99
siRNA (s.c.)	Phase I–II NCT03767829	Active, not recruiting Primary outcome: evaluate the safety and tolerability of single or multiple doses	100
Oral tablets	Phase II NCT01379469	Recruiting Primary outcome: determine the effect of carbamazepine on hepatic AAT polymers	101

AAT-AT, alpha-1 antitrypsin augmentation therapy; AAV, adeno-associated virus; AE, adverse event; COPD, chronic obstructive pulmonary disease; FEV1, forced expiratory volumen in 1 second; i.v., intravenous; NE, neutrophil elastase; RNAi, RNA interference; SAE, serious adverse event; s.c., subcutaneous; siRNA, small interfering RNA.

## Conclusion

Diagnostic techniques for AATD are improving, but milder genotypes (PiSZ and PiMZ) remain underdiagnosed in the general population. AAT-AT confers decreased emphysema progression and may need to be stopped prior to transplantation if disease progresses to this point. Whilst we can speculate that these potential benefits might be extended to milder forms like PiSZ, further investigations are still needed.
